# High Aspect Ratio Thin-Walled Structures in D2 Steel through Wire Electric Discharge Machining (EDM)

**DOI:** 10.3390/mi12010001

**Published:** 2020-12-22

**Authors:** Naveed Ahmed, Muhammad Ahmad Naeem, Ateekh Ur Rehman, Madiha Rafaqat, Usama Umer, Adham E. Ragab

**Affiliations:** 1 Industrial Engineering Department, College of Engineering and Architecture, Al-Yamamah University, Riyadh 11512, Saudi Arabia; naveed527@gmail.com; 2 Department of Industrial and Manufacturing Engineering, University of Engineering and Technology, Lahore 54890, Pakistan; ahmad.ravian@gmail.com (M.A.N.); madihanaveed100@gmail.com (M.R.); 3 Department of Industrial Engineering, College of Engineering, King Saud University, Riyadh 11421, Saudi Arabia; aragab@ksu.edu.sa; 4 Advanced Manufacturing Institute, College of Engineering, King Saud University, Riyadh 11421, Saudi Arabia; uumer@ksu.edu.sa

**Keywords:** wire electric discharge machining, machining, thin structures, deflection, D2 steel, fin thickness, fin length, fin height

## Abstract

Thin structures are often required for several engineering applications. Although thick sections are relatively easy to produce, the cutting of thin sections poses greater challenges, particularly in the case of thermal machining processes. The level of difficulty is increased if the thin sections are of larger lengths and heights. In this study, high-aspect-ratio thin structures of micrometer thickness (117–500 µm) were fabricated from D2 steel through wire electrical discharge machining. Machining conditions were kept constant, whereas the structure (fins) sizes were varied in terms of fin thickness (FT), fin height (FH), and fin length (FL). The effects of variation in FT, FH, and FL were assessed over the machining errors (FT and FL errors) and structure formation and its quality. Experiments were conducted in a phased manner (four phases) to determine the minimum possible FT and maximum possible FL that could be achieved without compromising the shape of the structure (straight and uniform cross-section). Thin structures of smaller lengths (1–2 mm long) can be fabricated easily, but, as the length exceeds 2 mm, the structure formation loses its shape integrity and the structure becomes broken, deflected, or deflected and merged at the apex point of the fins.

## 1. Introduction

Thin-walled structures are fundamental to a range of microfeatures required in various work materials [1]. The growing need for thin-walled structures is due to their capability for high heat dissipation and their light weight ratios [2]. Such structures are used widely in several engineering applications in the automotive, aerospace, biomedical, submarine, and other industries [3]. The high aspect ratio of thin structures is an additional requirement in such applications [4]. Thin microfeatures are fabricated by several manufacturing methods, including forming [5], additive manufacturing [6], welding [7], extrusion [8], investment casting [9] and machining, such as milling [10].

Various researchers employ micromilling to produce deep features within thin walls. However, the formation of burrs is a common limitation of micromilling. Burr formation occurs even in low aspect ratio structures (3:1), necessitating a secondary operation for burr removal. This increases the processing time and the production cost. Tool preparation is another limitation of the micromilling of thin features; if a tool wears out or a rupture occurs, further tool preparation becomes necessary [11]. To reduce the chances of tool failure, Xia et al. [4] employed laser-assisted micromilling to produce microgrooves in titanium alloy with an aspect ratio of 5.4. They reported a wall thickness of 500 µm using the laser-assisted milling.

Miranda et al. [12] used selective laser melting to fabricate thin plates of 0.3 mm width and 5 mm height (aspect ratio of ~17) from titanium alloy. Sudarsan et al. [13] used a stamping operation to create microchannels in ultra-thin steel sheets. They reported that they could produce microchannels of 1.5 mm width and 0.46 mm depth without any signs of deflection, buckling, and wrinkling. Electrochemical machining is another method that has been reported to produce microfins in copper. The geometry and accuracy of the fins remained under control for a low aspect ratio. The fabrication of long fins or channels has been reported as being challenging because of the difficulties of tool penetration and consequent accumulation of melt debris inside the machined features [14]. Laser machining is another commonly used process for producing thin walled structures; however, creating features with a high aspect is challenging. Oh et al. [15] used laser machining to produce microgrooves with an aspect ratio of 10 in stainless steel. However, Zhou et al. [16] stated that the fins or channels produced using laser beam machining are of low depth because of the difficulty in propelling of melt debris. Spatter dispersion during laser machining also affects the quality of machined features, in addition to that of the neighboring areas [17]. Abrasive water jet machining can be employed to produce structures with a high aspect ratio; however, the length and depth of such structures is limited because the features are damaged by high mechanical forces [18].

Wire electric discharge machining (WEDM) is a process that offers a huge cushion for fabricating complex features with good geometrical accuracy [19]. For example, Cheng et al. [20] used WEDM to manufacture a micromilling tool with a corner radius error of ±2 µm. The wires commonly used in the WEDM process are made of brass and molybdenum [21,22]. However, the use of molybdenum wire is more common in the cutting industry as compared to brass wire it offers a low wear rate and can be used repeatedly [23,24].

Different process responses and feature behavior has been reported for both thick and thin sections cut using WEDM [25]. The teeth of miniaturized gears can be considered to be thin structures in which it is important to retain a uniform and straight tooth geometry [26]. Liao et al. [27] reported that it is easier to fabricate single microfeatures using WEDM; however, if the structures are to be produced repeatedly in the form of structure arrays, the process becomes complicated and maintaining feature geometry is difficult. Hourmand et al. [28] used EDM to fabricate a microtool of 4.7 mm length that was used to produce a hole through die-sinking EDM. Zahiruddin et al. [29] reported that thermal deformation occurs in the thin structures produced through WEDM. The deformation curvature was found to be nonlinear due to the nonlinear distribution of thermal stresses. Zhange et al. [30] used WEDM to cut microbeams with an aspect ratio of 10 in tool steel. A high amount of deformation and deflection from the neutral axis has also been reported.

The literature also addresses the optimization of process parameters to achieve a definite length or depth of the machined feature [31]. Ahmed et al. [32] proposed an optimized set of WEDM parameters to produce large surface area microchannels in a copper substrate. They reported that straight fins of 939 µm length without deflection can be produced using the optimized parameters. In the case of using WEDM with tungsten carbide, the highest aspect ratio of 45 has been reported by [33] who prepared a microtool for die-sinking EDM. Azarsa et al. [34] proposed an optimized combination of abrasive water jet machining to produce thin structures in aluminum alloy. They produced free-standing fin structures, similar to the structures proposed in our present study. They machined the fins in two different plates of thicknesses 6 mm and 12 mm, which suggests that the fin heights (FHs) were 6 mm and 12 mm.

The literature reveals that the formation or machining of thin structures is of great importance in the manufacturing industry. Machining processes are used to produce thin structures, and optimized parameters are recommended to produce structures of good quality. Achieving a high aspect ratio is the common concern of machinists. We were unable to find any studies discussing the effect of variation in feature length, thickness, and height, particularly for thin structures with a high aspect ratio produced using WEDM. In this study, we produced free-standing thin structures (microthickness fins) from D2 steel using WEDM. We first identified the optimized parameters for cutting D2 steel and then produced the thin structures under the constant machining conditions of molybdenum wire EDM. The novelty of this study is that we have investigated the effect of varying sizes of free standing fins over the fin quality and machining errors while keeping the machining parameters at constant levels. To achieve values of minimum thickness and maximum length and height the fins are produced with a thickness variation of 1–0.3 mm, a height variation of 10–30 mm, and a length variation of 1–40 mm. Thin structures with a straight and uniform profile and no deflections can be produced with an aspect ratio of 40 (20 mm length and 500 µm thickness) for FHs of 10–30 mm. However, regardless of FH, structures with an aspect ratio of 80 (40 mm length and 500 µm thickness) exhibit a slight deflection at their free ends.

## 2. Materials and Methods

**Figure 1:**
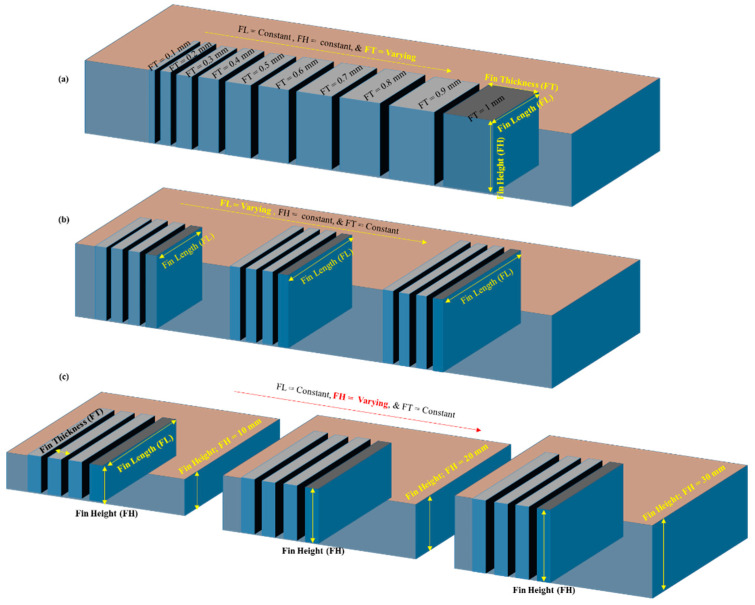
Schematic of fin structures: (**a**) varying fin thickness, (**b**) varying fin length, and (**c**) varying fin height.

We chose D2 steel as the work material for cutting thin structures through WEDM. As EDM is a process that not only depends on the machining parameters but also is one in which the performance is a function of thermoelectric properties of the material to be cut, the elemental composition and the important properties of D2 steel are shown in Table 1 and Table 2, respectively. Thin structures, known as “fins,” of various sizes were produced. A fin has three conventional dimensions: fin thickness (FT), fin length (FL), and FH. We have taken these dimensions to be the research variables. We varied each of these three fin dimensions to study the fin quality. FT was varied from 1 mm to 0.3 mm in equal increments of 0.1 mm from the preceding value. This produced seven levels of FT. Similarly, we produced seven levels of FL, with each new FL being larger than the preceding FL. For example, we produced five FLs of 2, 4, 6, 8, and 10 mm at increments of 2 mm. For longer fins, we then doubled the length, e.g., 20 mm and 40 mm. We took FH at three levels: 10 mm, 20 mm, and 30 mm. We used three workpieces of different thicknesses. Therefore, as shown schematically in Figure 1, FHs of 10, 20, and 30 mm were the workpiece thicknesses. We evaluated machining performance in terms of three response measures: the FT error (FT_e), the FL error (FL_e), and the fin quality. Table 3 shows all of these variables and responses, in addition to the machining parameters. The FT_e is the difference between the designed values and actual machined values of FT. Similarly, the FL_e is the difference between the designed and actual machined lengths. Fin quality varies as the fin dimensions are varied. Our aim was to obtain fin structures with minimum possible thickness and maximum possible length without any damage in respect of the straightness and cross-section of the structures. We observed damage in terms of deflection, ruptures at the top end, and fins that were straight at the base but were deflected and merged at the top. For ease of analysis, we classified and coded the fin quality characteristics numerically. The classification and the corresponding codes are shown in the last column of Table 3.

**Figure 2:**
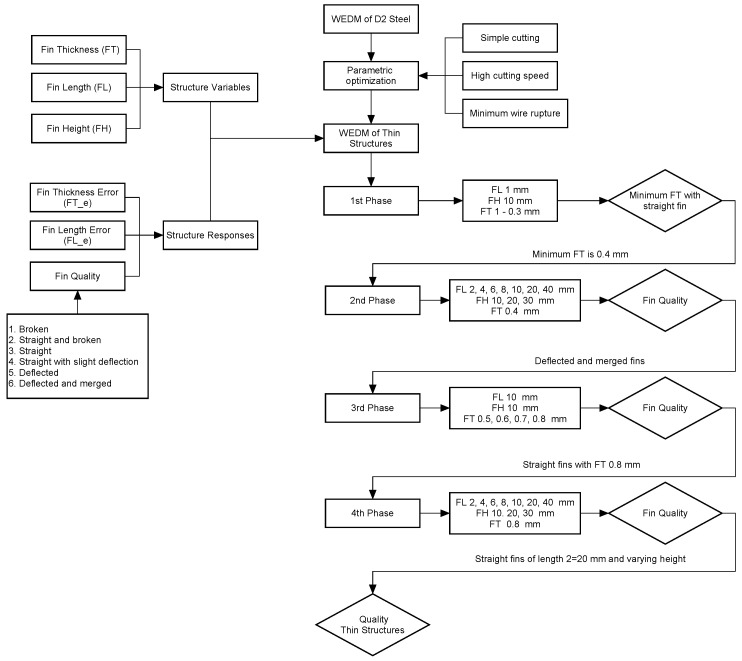
Research methodology to fabricate high aspect ratio thin structures in D2 steel through wire electric discharge machining (WEDM).

**Figure 3:**
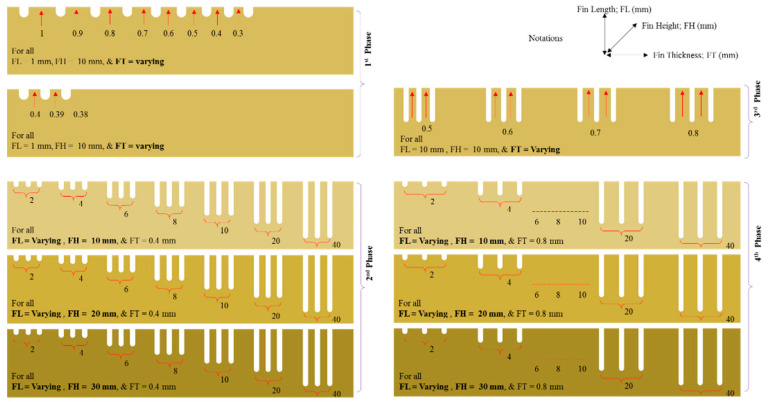
Layout of high aspect ratio thin structures with respect to phased experimentation (dimensions are not to scale).

As our aim was to evaluate the effect of fin dimensions over the quality of fin structures, we produced all the structures under constant machining conditions, as shown in Table 3. Before we fabricated the fins, we conducted several trial experiments on D2 steel to make simple cuts. We sought to identify parameters that would result in high cutting speed and minimum wire rupture. We kept these machining parameters constant throughout the experimentation. In this way, the effects of WEDM parameters on the fin quality were minimized and unified. The experimentation was conducted in a phased manner. Figure 2 shows the research methodology, and Figure 3 presents the schematic of the phased experimentation. In the first phase of experimentation, fins were produced from a work plate of 10 mm (FH 10 mm). During this phase, an FL of 1 mm was kept constant. The only variable was FT, which started at 1 mm. After each cut, we reduced the FT gradually, in 0.1 mm increments, until it was no longer possible to achieve a thickness value. It can be said that the first phase of experimentation is the screening of FT that can possibly be fabricated in D2 steel. We took the FT value that we obtained after the first phase was taken as the input of the second phase. In the second phase, FT was kept at constant level and FL was varied, as shown in Table 3. From the 10 mm–thick work plate, we produced seven fins of 2, 4, 6, 8, 10, 20, and 40 mm in length. We repeated production of the same set of fins in the second and third plates of 20 mm thickness (FH 20 mm) and 30 mm thickness (FH 30 mm), respectively. The observations from the second phase led to the third phase of experimentation, in which FL and FH were kept at constant levels and FT was varied again. This enabled further fine turning of the FT was carried out based on the fin quality characteristics. A designed FT (FT_d) of 0.8 mm was considered to be the input of the fourth phase of experimentation. The fourth phase of experimentation follows the same pattern as in the case of 2nd phase. The only difference is the FT. The flowchart in Figure 2 and the detailed schematic of the phase-wise experimentation in Figure 3 may help in understanding the experimental scheme.

We measured the machined fins using a coordinate measuring machine) with a measurement resolution of 1 µm. FT was measured at three points on each fin, and the average values were recorded. The difference between the designed and the machined thickness results in FT_e. Similarly, we measured the FLs, and the difference between the designed and machined lengths is FL_e. It must be noted that two fins were produced in each experimental run to ensure repeatability of the machining and measurement.

## 3. Results and Discussion

**Figure 4:**
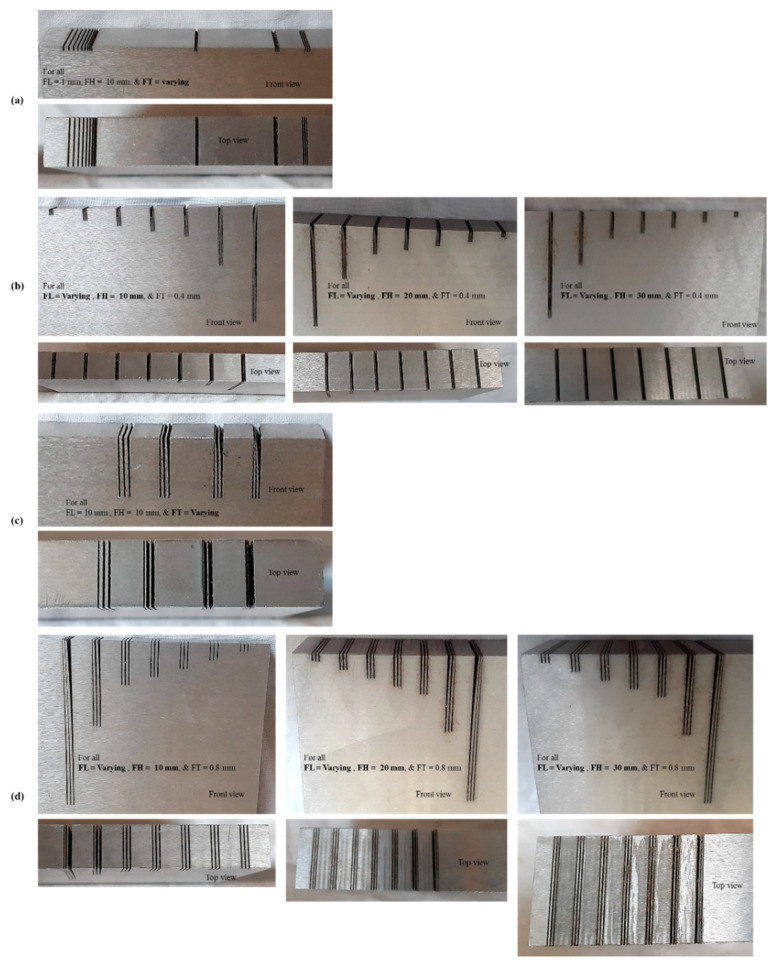
Actual fin structures fabricated on different plates of D2 steel through WEDM. Structures from (**a**) first phase, (**b**) second phase, (**c**) third phase, and (**d**) fourth phase of experimentation.

Following the experimentation under constant machining conditions we conducted measurement and analysis. Table 4 shows the experimental results pertaining to each of the four phases of experimentation. They consist of FH, FT_d, designed FL (FL_d), machined FT (FT_m), machined FL (FL_m), FT_e, FL_e, fin quality (classification), and fin quality codes. Figure 4 shows thin structures (fins) produced in different work samples of D2 steel using WEDM, with front and top views provided. The produced fins can be seen to be either straight, deflected, or straight with deflection at the free end. However, some of the fins were broken at the free end. We performed microscopic analysis of each of the fin structures. Moreover, we have graphically presented the effect of varying feature sizes over the machining error and fin quality. The subsequent sections categorically discussed the detailed analysis of each of the experimental phases.

### 3.1. Analysis of First Phase

**Figure 5:**
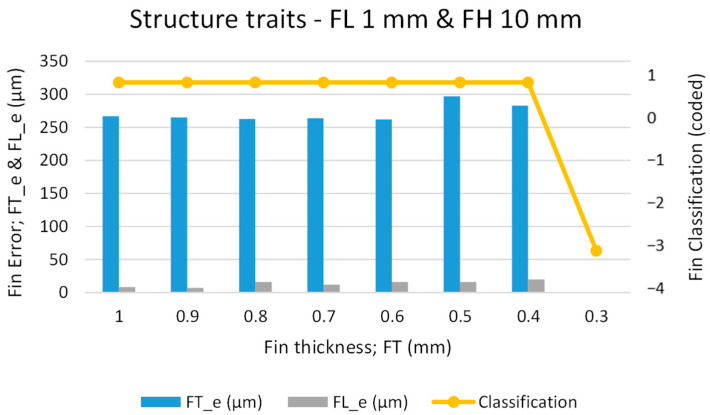
Graphical analysis of structure traits in the first phase of experimentation.

**Figure 6:**
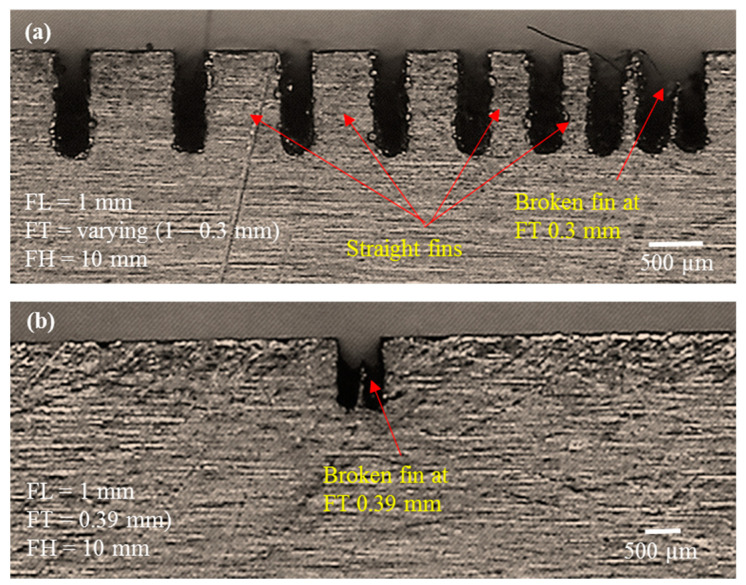
Microscopic analysis of structure traits in the first phase of experimentation. (**a**) fins with varying thickness but with constant length and height; (**b**) broken fin with thickness less than 0.4 mm.

As the first phase of experimentation was to screen for the minimum possible FT of fins to be produced, our main criterion was to produce straight fins with minimum possible thickness. During the first phase, FL was kept constant, at a very small value of 1 mm. However, we stepped the FT down from 1 mm to 0.3 mm at step intervals of 0.1 mm. We kept the FH at a constant value of 10 mm (work plate thickness). Figure 5 presents our graphical analysis of FT_e, FL_e, and fin classification. The primary vertical axis represents machining errors whereas the secondary axis represents the fin classification, coded as per Table 3. One can that the fins remain straight (straight fins are coded as “1”) as the thickness is reduced from 1 mm to 0.4 mm. However, as can be seen from microscopic images in Figure 6, at a designed thickness of 0.3 mm, fins that were broken at the top end were produced. A broken fin is coded as “−3” (for coding, refer to Table 3). We extended the screening further to create fins of 0.39 mm and 0.38 mm thickness; however, as shown in Figure 6b, the fabricated fins were broken. Thus, we can infer that 0.4 mm is the suitable designed fin thickness (FT_d) to achieve straight fins without rupture or deflection.

With reference to machining error in fin thickness (FT_e), one of the main reasons is the inappropriate selection of the offset during WEDM. In this study, zero offset is taken for all the experiments and thus the error caused by inappropriateness of offset can stay at the constant level. Another reason is the fin thickness to be produced. The larger the fin thickness is, the more available material to absorb and distribute thermal energy. In this way the energy density is low and a controlled amount of material removal is achieved. On the other hand, for small fin thicknesses the energy density is high due to the availability of less amount of work material under electric discharges. Therefore, excessive material is removed and consequently more error is resulted as compared to thick fins. As it can be seen from Figure 5, the error remains uniform at ~250 µm when FT is set from 1 mm to 0.6 mm. The error slightly jumps at ~300 µm when the FT value passes 0.6 mm and reaches 0.4 mm. This FT_e is the undercut caused by the kerf width due to offsetting associated with the WEDM process. As the machining conditions were kept constant, zero offset was taken throughout the experimentation to maintain the uniformity of the machining results. Conversely, FL_e was found to be very small compared to FT_e. The reason behind this is the extremely high values of fin length (multiple millimeters) as compared to fin thickness (1 or less than 1 mm). The difference or error in material removal is insignificant with respect to length whereas the difference or error is noticeable in case of thickness. However, as the FT is reduced the FL_e tends to increase slightly. This increase in FL_e is the result of either the deflection that occurs during fin formation or the fin breakage at the tip (overall length reduction). Thus, we have found that thin structures of 0.4 mm designed thickness (and ~150 µm actual thickness) can be successfully produced if the FL is selected as 1 mm.

### 3.2. Analysis of Second Phase

After the first phase of experimentation, we identified 0.4 mm as the suitable FT_d to machine 1 mm long fins with a height of 10 mm. What about the fin quality if the FL and FH are increased? To answer this question, we designed the second phase of experimentation, in which the designed value of FT was kept constant at 0.4 mm whereas the FHs and FLs were varied. This variation in two aspects (length and height) was taken one at a time. For example, in the first step of the second phase, we kept FT and FH constant at 0.4 mm and 10 mm, respectively, and only varied FL in the sequence of 2, 4, 6, 8, 10, 20, and 40 mm (as shown in Figure 3). Similarly, in the second step, we took an FT of 0.4 mm and an FH of 20 mm as constant and varied FL. We repeated this pattern in the third step, with an FT of 0.4 mm and an FH of 30 mm.

**Figure 7:**
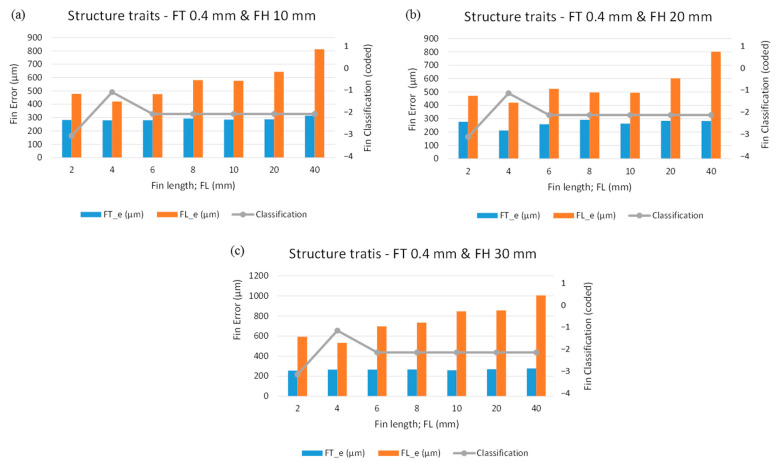
Graphical analysis of structure traits under the second phase of experimentation with designed fin thickness (FT_d) of 0.4 mm: (**a**) FH 10 mm, (**b**) FH 20 mm, and (**c**) FH 30 mm.

Figure 7 graphically illustrates the effect of varying fin sizes over the machining errors and fin classification. Figure 7a displays the effect of variation in FL over the machining responses when the FT and FH are 0.4 mm and 10 mm, respectively. One can see that the FT_e remains almost the same for the entire range of FL. As shown in Table 4, the FT_e fluctuates within 280–290 µm until the FL reaches 20 mm. However, the FT_e exceeds 310 µm when a 40 mm long fin is produced. On the other end, we found the machining FL_e to be 480 µm against an FL of 2 mm. At 2 mm FL, we found the fin classification to be coded as “−3”, indicating that the fin is broken at the top end resulting in length reduction and high machining error. As the FL reaches 4 mm the structure of the fin is not broken, but the fin is deflected at the top end. That is why the value of FL_e is reduced and the classification code reaches “−1” (deflection code). Beyond an FL of 6–40 mm (long fins) the FL_e increases continuously (Figure 7a), and all these FLs acquire a classification code of “−2”, indicating that the fins are both deflected and merged at the free ends. As shown in Figure 7b,c, similar behavior was noticed when the FLs were varied in the cases of 20 mm FH and 30 mm FH. Hence, the machining responses are prone to FL, and there is no considerable effect of FH over the machining responses.

**Figure 8:**
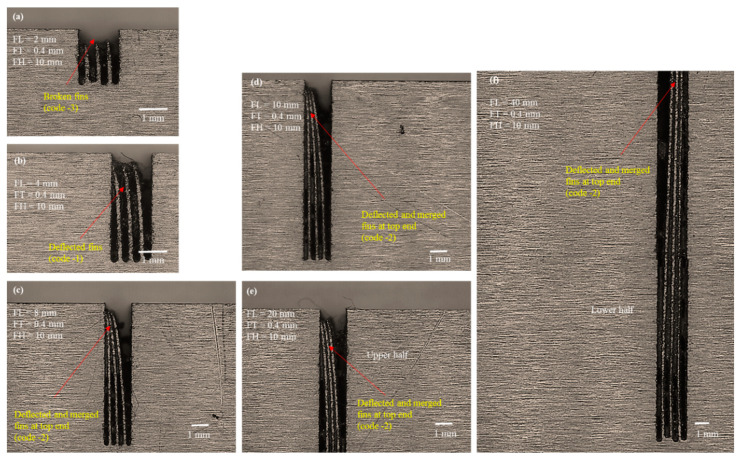
Microscopic analysis of structure traits under the second phase of experimentation with designed fin thickness (FT_d) of 0.4 mm and FH 10 mm: (**a**) FL 2 mm, (**b**) FL 4 mm, (**c**) FL 8 mm, (**d**) FL 10 mm, (**e**) FL 20 mm upper half, and (**f**) FL 40 mm lower half.

**Figure 9:**
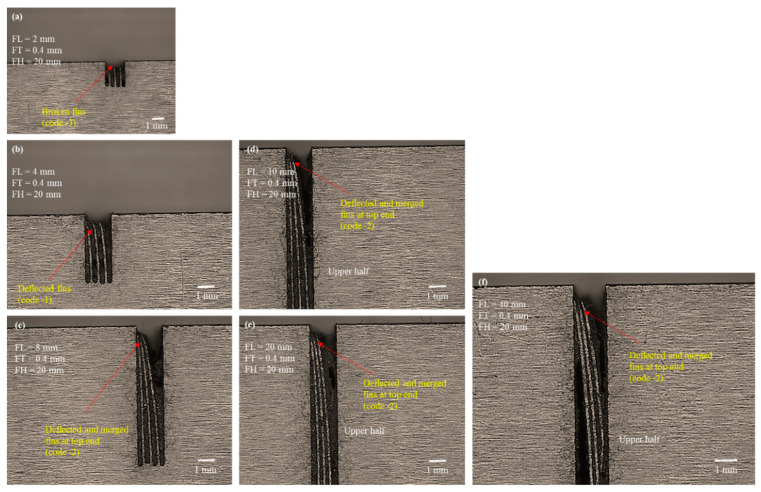
Microscopic analysis of structure traits under the second phase of experimentation with designed fin thickness (FT_d) of 0.4 mm and FH 20 mm: (**a**) FL 2 mm, (**b**) FL 4 mm, (**c**) FL 8 mm, (**d**) FL 10 mm upper half, (**e**) FL 20 mm upper half, and (**f**) FL 40 mm upper half.

**Figure 10:**
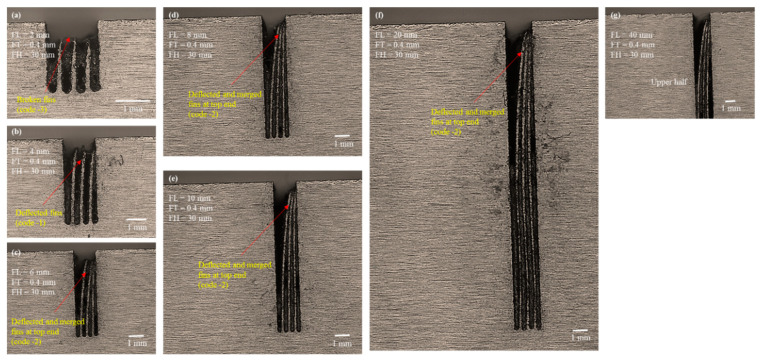
Microscopic analysis of structure traits under the second phase of experimentation with designed fin thickness (FT_d) of 0.4 mm and FH 30 mm: (**a**) FL 2 mm, (**b**) FL 4 mm, (**c**) FL 6 mm, (**d**) FL 8 mm, (**e**) FL 10 mm, (**f**) FL 20 mm, and (**g**) FL 40 mm upper half.

We conducted microscopic analysis to elucidate the reason for the said behavior. Figure 8, Figure 9 and Figure 10 show the selected microscopic images of the machined structures produced with different FHs and FLs. Figure 8 depicts 10 mm FH. Figure 8a shows that the machined structures with 2 mm designed length were straight, though the actual length was less than the designed length. Fins were broken at the top. The FT_d was kept constant at 0.4 mm, whereas the thickness of the actual machined fins was approximately 120 µm (~280 µm is the machining FT_e). When the wire completes its travel on the left-hand side of the fin, the structure is thick (solid block at the right-hand side) and the discharge energy dissipates toward the thick side of the cut. As the wire begins its travel to the right-hand side of the previous cut, the structure tends to become thin and fin formation is initiated. At this point, the discharge energy produced under the electric discharges must be dissipated toward the moving wire and the thin portion of the formed structure (~120 µm thin). Under the high temperature of discharge energy, the thin portion at the top end begins to melt during cutting. In this way, a needle-like tip is produced at the top end, as shown in Figure 8a. This phenomenon at the apex of the fins is unchanged when FL is further increased, as shown in Figure 8b, where the FL is 4 mm. The fins are also melted and broken at the tip.

During the process of electric erosion, a plasma plume or a plasma channel with high pressure and temperature (>10,000 °C) is produced inside the localized sparking area. Thus, the workpiece being machined experiences high plasma pressure [37], which produces micro/nanoforces (non-macroforces) [30]. The plasma pressure and forces are shared by the wire electrode and the machined feature. The effects of such pressure and forces may be negligible when machining a thick feature. However, this effect is significant if the feature to be machined is a thin feature of micrometer thickness, and it produces geometrical inaccuracies and structural bending due to thermal deformation [38]. The bending phenomenon becomes more prominent as the length of the structure is increased further. When the FL is increased from 2 to 4 mm, an individual fin acts like a hanging and free-end structure (like a cantilever beam). Thus, under the action of microforces and plasma plume pressure the extended structure is deflected at the upper end. As the machined structure also experiences localized heating and softens, there is a slight deflection at the thin, soft, and extended region of the fin. Miller et al. [39] cut thin cross-sections in pure titanium using WEDM and reported that the cut sections bent noticeably at the upper end. Similar results were observed when the FLs were increased further from 4 mm to 40 mm (Figure 8c–f). As the length increased, the deflection became more prominent. Each of the fabricated thin structures of 6, 8, 10, 20, and 40 mm in length were deflected at the upper part and merged at the apex. We have termed this classification of structures as “deflected and merged” and coded it as “−2”. Each of these fins remained straight at the bottom end but tended to deflect and merge at the point where fin formation started. We observed similar behavior when cutting these structures with FHs of 20 mm and 30 mm. The selected microscopic images associated with FH 20 mm and FH 30 mm are shown in Figure 9 and Figure 10, respectively. The results suggest that the formation of thin structures is not seriously dependent on the FH but, rather, depends on the FT and the FL. The results of the second phase of experimentation, suggest that 0.4 mm FT_d is not a perfect choice if the aim is to produce high aspect ratio thin structures of more than 1 mm in length.

### 3.3. Analysis of the Third Phase

**Figure 11:**
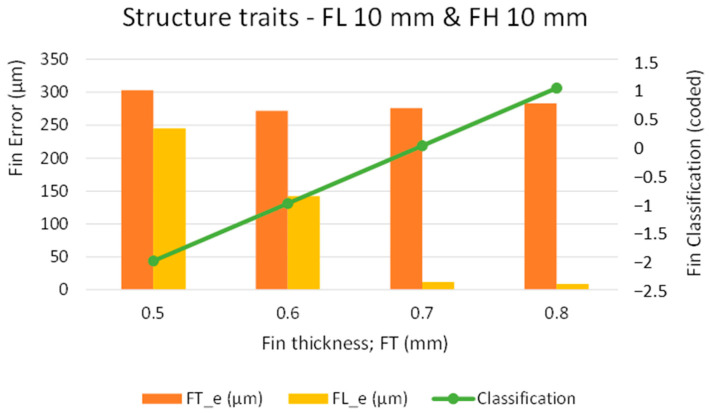
Graphical analysis of structure traits in the third phase of experimentation.

**Figure 12:**
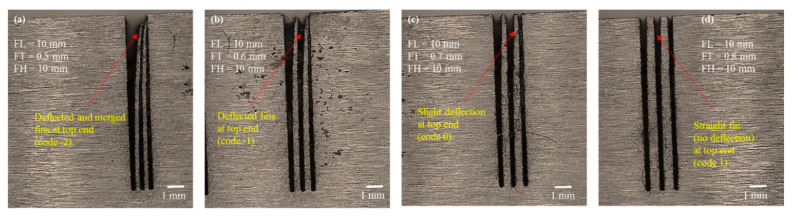
Microscopic analysis of structure traits in the third phase of experimentation, with FL 10 mm and FH 10 mm: (**a**) FT_d 0.5 mm, (**b**) FT_d 0.6 mm, (**c**) FT_d 0.7 mm, (**d**) FT_d 0.8 mm.

As we found the fins with a designed thickness of 0.4 mm to be broken at the top end and observed deflection, an FT_d of 0.4 mm is not suitable, particularly for high aspect ratio and long fin structures (FL more than 2 mm). Therefore, we carried out the third phase of experimentation to further screen out and tune the most suitable FT_d. As the problem with the structures is more serious in long fins, in this phase of the experimentation, we conducted the screening of FT for fins of 10 mm in length. The FT_d was varied from 0.5 mm until we could obtain straight fins without any deflection. Figure 11 present the experimental results graphically, and Figure 12 shows the microscopic images of the fabricated fins. As can be seen from Figure 11, when the FT_d is set at 0.5 mm, the error in FT is 300 µm and the error in FL is 240 µm. This indicates that the fins were again broken at the top and are also merged at the tip (as also shown in Figure 12a). For this reason, both errors were found to be high. The corresponding classification code is “−2” which is equivalent to “deflected and merged fins.” As the fin thickness was increased to 0.6 mm, there was a noticeable reduction in the values of both FT_e and FL_e. However, the fins were again bent at the top, as shown in Figure 12b. The corresponding classification code is “–1” which is equivalent to “deflected fins.” With 0.7 mm-thick fins, there was an abrupt reduction in FL_e. An error of just 12 µm was obtained in FL. However, the value of FT_e remained almost unchanged (~270 µm), due to the undercut and the nonprovision of offsetting. As shown in Figure 12c, we obtained straight fins with only slight deflection (classification code “0”). This phenomenon of slight deflection vanished completely when we selected the FT_d of 0.8 mm. In this case, the value of FL_e was also further reduced. As shown in Figure 12d, we obtained straight fins without any deflection (classification code “1”).

It must be noted that the machined fin thickness (FT_m) was 0.51 mm (~510 µm) against the FT_d of 0.8 mm. Hence, we can state that an FT_d of 0.8 mm is the most suitable thickness for obtaining straight thin structures (~510 µm thickness) without any evidence of rupture, deflection, and merging at the entry point of the fin. So far, the above statement holds true for 10 mm long fins with an aspect ratio of 20. Thus, in the fourth phase of experimentation, we tested the suitability of the identified FT of 0.8 mm for fins longer than 10 mm and with an aspect ratio of greater than 20.

### 3.4. Analysis of the Fourth Phase

**Figure 13:**
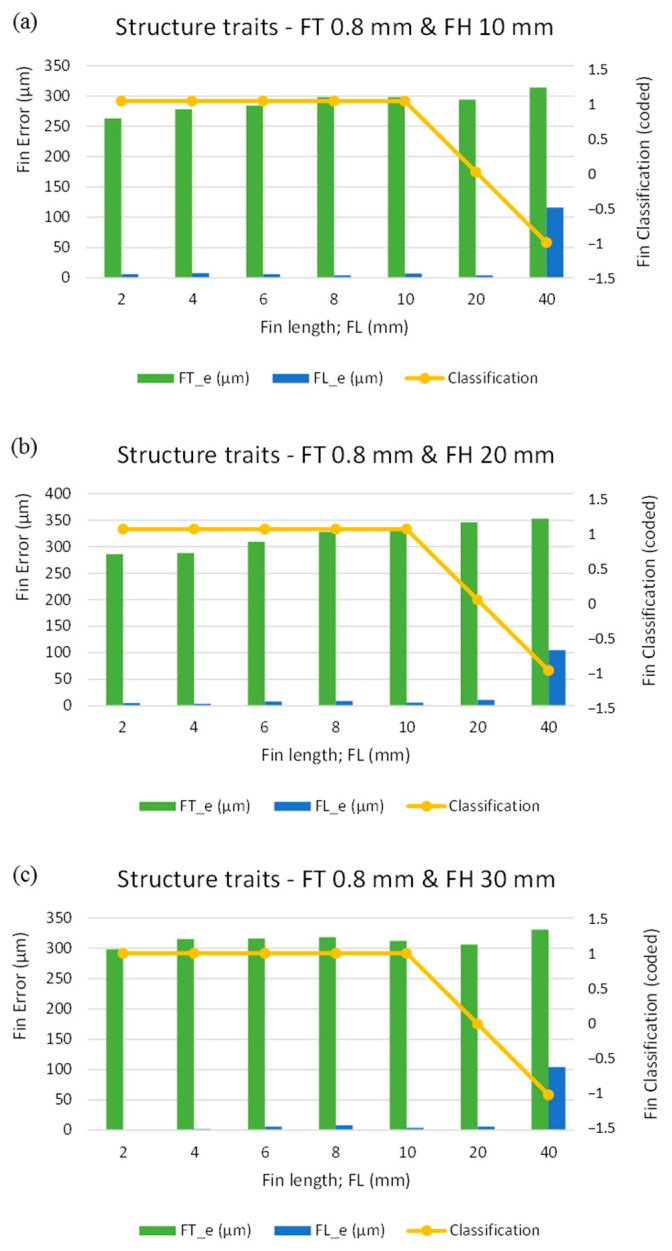
Graphical analysis of structure traits in the fourth phase of experimentation with designed fin thickness (FT_d) of 0.8 mm: (**a**) FH 10 mm, (**b**) FH 20 mm, and (**c**) FH 30 mm.

In the third phase, we found an FT_d of 0.8 mm to be the most suitable value for fabricating thin and long fins from 10 mm thick plate (FH 10 mm) without any rupture and deflection. To validate the response of the said fin thickness (FT_d 0.8 mm), the fourth phase involved for several structure lengths, ranging from 2 mm to 40 mm. We performed seven experiments on 10 mm thick plate (FH 10 mm) and repeated the same set of experiments with 20 mm and 40 mm thick plates (FH 20 mm and 30 mm). Figure 13 graphically presents the machining results. As can be seen, with the increase in FL, there was a slight increase in FT_e, though the error in FT remained between 260 µm and 310 µm. As discussed in previous sections, the source of this machining error has been identified as the undercut caused by zero offset setting. However, as the FH was increased from 10 mm to 20 mm the values of FT_e approached 350 µm (Figure 13b). As the FH is increased, the contact area of the work surface and wire increases. Larger interacting contact areas allow discharges to occur and spread over a greater area, causing high erosion. In this way, the thickness of the machined structures decreases as compared to the designed thickness. Ultimately, this generates relatively more machining errors. Thus, against the FT_d of 0.8 mm, the machined FT was observed to be 0.5 mm (500 µm), on average. With respect to the FL_e, the difference between the FL_d and the FL_m was found to be very small, amounting to approximately 10 µm, as shown in Figure 13. For the lengths of 2–20 mm, the value of FL_e fluctuated around 10 µm; however, in fins of 40 mm length a noticeable length reduction, approaching 100 µm, was attained. This difference in actual FL_m was caused by the deflection observed in fins of 40 mm Length. That is the reason why the trend line of the fin classification sinks from a constant horizontal line (straight fin with code “1”) and ends at “−1”, representing the deflected fin. The trends in FT_e, FL_e and fin classification follow a similar pattern for each of the three FHs (10, 20, and 30 mm).

**Figure 14:**
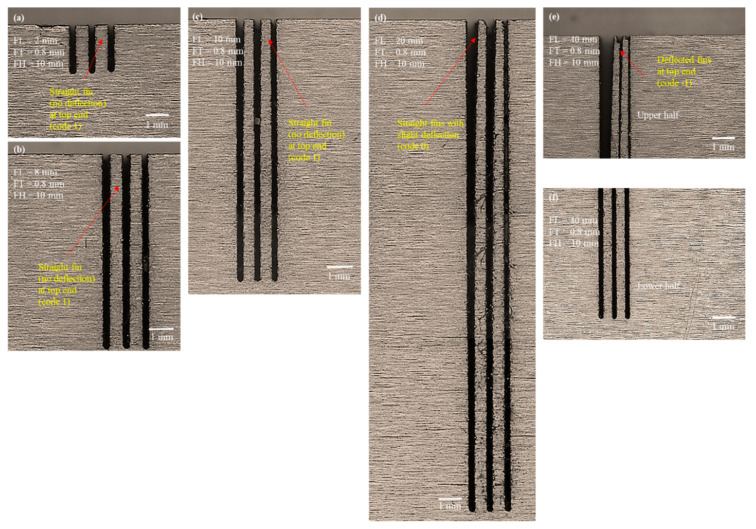
Microscopic analysis of structure traits in the fourth phase of experimentation with designed fin thickness (FT_d) of 0.8 mm and FH 10 mm: (**a**) FL 2 mm, (**b**) FL 8 mm, (**c**) FL 10 mm, (**d**) FL 20 mm, (**e**) FL 40 mm upper half, (**f**) FL 40 mm lower half.

We conducted microscopic analysis of the machined structures for each of the seven fins produced in each of the three plates. The results were similar irrespective of FH. Therefore, Figure 14 shows only the microscopic images of 10 mm thick plate. As can be seen from Figure 14a–c, fin structures of lengths 2–10 mm were perfectly straight and showed no sign of rupture, deflection, and merged deflection, as was the case when the same-sized structures were produced with an FT_d of 0.4 mm. We also found the cross section of each of the fins to be uniform throughout the length. When the FL reached 20 mm, we observed a minor deflection at the tip of the fin, as can be seen in Figure 14d. However, for exceptionally long fins of 40 mm length, the fins remained straight from the bottom to above midlength, although they tended to deflect in the upper half. Figure 14e shows the upper halves of 40 mm long fins, and Figure 14f shows the lower halves. This deflection was entirely different from the deflection observed in previous cases of 0.4 mm thin structures, wherein the long fins were deflected and merged at the top end. However, at 0.8 mm FT_d the deflection did not merge in any case, not even for 40 mm long fins (aspect ratio of 80). Moreover, such a small deflection can be rectified by combing, if required. One can also note that we found the actual thickness of the machined structures to be almost 0.5 mm lower than the designed thickness of 0.8 mm. Thus, we can infer that the long thin structure of 500 µm thickness with the highest aspect ratio of 80 can be obtained successfully without structure breakage, conjunction, bending, and deflection. In this case, the cross sections of the free standing ends also maintained a uniform square shape without the formation of any needlelike ends as were observed the second phase of the experimentation. Ali and Muhammad [40] reported similar results, albeit for low aspect ratio structures (aspect ratio 3). They developed a copper microtool and gear comprising a repeated number of fin blades. They reported low-height blades of 1.5 mm height and 500 µm thickness cut by WEDM.

## 4. Conclusions

Thin structures (long fins) of micrometer thickness with a high aspect ratio have been produced in D2 steel through wire electrical discharge machining under constant machining conditions. Fin thickness (FT) varied from 1 mm to 0.3 mm, fin height (FH) varied from 10 mm to 30 mm, and fin length (FL) varied from a smaller value of 1 mm to an exceptionally larger value of 40 mm. The structures have been evaluated in terms of FT error (FT_e), FL error (FL_e) and fin quality (straight fins with minimum deflection and machining errors). Based on the results, discussion, and graphical and microscopic analyses, we have drawn the following important inferences:

- Fabrication of thin structures through WEDM is possible, but the structure formation largely depends on its thickness and length provided that the WEDM conditions remained constant. Thin structures are relatively more difficult to fabricate as compared to thick structures (FT > 600 µm). It is even more difficult to fabricate thin structures if the structure length is increased i.e., to have high aspect ratio structures.
- On the whole, the formation of thin structures takes four forms. The structures are either: (1) broken at the tip point with needle-like spikes, (2) largely deflected, (3) deflected and merged at the upper ends, (4) slightly deflected, and (5) perfectly straight with square ends.
- With zero offset, constant machining conditions (as used in this study), and 1 mm structure length (FL 1 mm), the minimum designed thickness of the structure to ensure that it is straight without deflection is 0.4 mm, and the corresponding actual machined fins are 117 µm thick (aspect ratio of 8.5).
- For structures longer than 1 mm under the designed fin thickness (FT_d) of 0.4 mm, the fins are broken at the apex and a sound thermal deflection is experienced. The deflection is proportional to the FLs. Fins longer than 4 mm not only deflect but also merge at their free standing ends.
- Under the FT_d of 0.8 mm with zero offset and constant machining conditions (as used in this study), approximately 500 µm thin structures can be produced successfully from D2 steel (aspect ratio of 80). Under this designed fin thickness, the structures are:
  - Straight and uniform throughout the length, if the FL ≤ 10 mm;
  - Slightly deflected at the top end, if the FL is 10 mm < FL ≤ 20 mm;
  - Straight at the lower half and deflected at the upper half, if the FL ≥ 40 mm.
- The microscopic evidence for each of the three FHs (10, 20, and 30 mm) are similar, irrespective of whether the fins are broken, deflected, or straight. Thus, the formation of thin structures (cross-section) is independent of their heights (FH) during the WEDM of D2 steel plates of 10–30 mm thickness.
- In general, fins longer than 20 mm experience relatively more machining errors (FT_e and FL_e) as compared to errors associated with fins of less than 20 mm in length. However, more specifically:
  - The error in fin thickness (FT_e) increases with the increase in fin length (FL) and fin height (FH).
  - The error in fin length (FL_e) only increases with the increase in fin length (FL). The fin height (FH) does not show a considerable effect on the fin length error (FL_e).

## Figures and Tables

**Table 1:** Elemental composition of D2 steel [35].

**Elements**	**C**	**Si**	**Mn**	**Mo**	**Cr**	**Ni**	**V**	**Co**	**Fe**
**Contents %**	1.5	0.3	0.3	1	12	0.3	0.8	1	Balance

**Table 2:** Physical, mechanical, and thermal properties of D2-grade tool steel [36].

Physical Properties		Mechanical Properties		Thermal Properties	
Properties	Value (Units)	Properties	Value (Units)	Properties	Value (Units)
Density	7.7 × 1000 kg/m^3^	Hardness, Rockwell C	62	Thermal expansion (20 °C)	10.5 × 10^−6^ °C^−1^
Melting point	1421 °C	Hardness, Vickers	748	Thermal conductivity	20 W/mK
		Poisson’s ratio	0.27–0.30		
		Elastic modulus	190–210 GPa		

**Table 3:** Constant machining conditions, variables, and responses set to cut thin structures in D2 steel.

**Constant Machining Conditions**	**Parameter**	**Value**							
	Machine	EDM E-7735							
	Wire	Molybdenum							
	Wire diameter (mm)	0.2							
	Discharge current; I (A)	2							
	Spark voltage (V)	160							
	Wire feed	37							
	Pulse on time (µs)	40							
	Pulse off time (µs)	40							
**Variables**	Variable name	Levels							
	Fin thickness; FT (mm)	1	0.9	0.8	0.7	0.6	0.5	0.4	0.3
	Fin height; FH (mm)	10	20	30					
	Fin length; FL (mm)	1	2	4	6	8	10	20	40
**Responses**	Response name								
	Fin thickness error; FT_e (µm)								
	Fin length error; FL_e (µm)								
	Fin quality	Classification							Code
		Straight							1
		Straight with slight deflection							0
		Deflected							−1
		Deflected and merged at the top end							−2
		Straight but broken at the top end							−3
		Broken							−4

**Table 4:** Experimental results of thin structures produced in D2 steel.

Exp. No	FH (mm)	FT_d (mm)	FL_d (mm)	FT_m (mm)	FL_m (mm)	FT_e (µm)	FL_e (µm)	Classification and Code	
**First Phase**									
**1**	10	1	1	0.733	0.992	267	8	Straight	1
**2**		0.9		0.635	0.993	265	7	Straight	1
**3**		0.8		0.537	0.984	263	16	Straight	1
**4**		0.7		0.436	0.988	264	12	Straight	1
**5**		0.6		0.338	0.984	262	16	Straight	1
**6**		0.5		0.203	0.984	297	16	Straight	1
**7**		0.4		0.117	0.98	283	20	Straight	1
**8**		0.3						Straight and broken	−3
**9**		0.39						Broken	−4
**10**		0.38						Broken	−4
**Second Phase (FH 10 mm)**									
**1**	10	0.4	2	0.118	1.522	282	478	Straight and broken	−3
**2**			4	0.12	3.579	280	421	Deflected	−1
**3**			6	0.119	5.524	281	476	Deflected and merged	−2
**4**			8	0.107	7.42	293	580	Deflected and merged	−2
**5**			10	0.115	9.424	285	576	Deflected and merged	−2
**6**			20	0.113	19.358	287	642	Deflected and merged	−2
**7**			40	0.086	39.188	314	812	Deflected and merged	−2
**Second Phase (FH 20 mm)**									
**1**	20	0.4	2	0.123	1.529	277	471	Straight and broken	−3
**2**			4	0.188	3.58	212	420	Deflected	−1
**3**			6	0.141	5.476	259	524	Deflected and merged	−2
**4**			8	0.109	7.503	291	497	Deflected and merged	−2
**5**			10	0.137	9.505	263	495	Deflected and merged	−2
**6**			20	0.116	19.398	284	602	Deflected and merged	−2
**7**			40	0.117	39.199	283	801	Deflected and merged	−2
**Second Phase (FH 30 mm)**									
**1**	30	0.4	2	0.145	1.407	255	593	Straight and broken	−3
**2**			4	0.135	3.467	265	533	Deflected	−1
**3**			6	0.135	5.304	265	696	Deflected and merged	−2
**4**			8	0.133	7.266	267	734	Deflected and merged	−2
**5**			10	0.141	9.154	259	846	Deflected and merged	−2
**6**			20	0.131	19.145	269	855	Deflected and merged	−2
**7**			40	0.123	38.996	277	1004	Deflected and merged	−2
**Third Phase**									
**1**	10	0.5	10	0.197	9.755	303	245	Deflected and merged	−2
**2**		0.6		0.328	9.858	272	142	Deflected	−1
**3**		0.7		0.424	9.988	276	12	Slight Deflection	0
**4**		0.8		0.517	9.991	283	9	Straight	1
**Fourth phase (FH 10 mm)**									
**1**	10	0.8	2	0.537	1.994	263	6	Straight	1
**2**			4	0.522	3.992	278	8	Straight	1
**3**			6	0.516	5.994	284	6	Straight	1
**4**			8	0.502	7.996	298	4	Straight	1
**5**			10	0.502	9.993	298	7	Straight	1
**6**			20	0.506	19.996	294	4	Straight (Slight Deflection)	0
**7**			40	0.486	39.884	314	116	Deflected	−1
**Fourth phase (FH 20 mm)**									
**1**	20	0.8	2	0.514	1.995	286	5	Straight	1
**2**			4	0.512	3.996	288	4	Straight	1
**3**			6	0.491	5.992	309	8	Straight	1
**4**			8	0.472	7.991	328	9	Straight	1
**5**			10	0.466	9.994	334	6	Straight	1
**6**			20	0.454	19.989	346	11	Straight (Slight Deflection)	0
**7**			40	0.447	39.895	353	105	Deflected	−1
**Fourth phase (FH 30 mm)**									
**1**	30	0.8	2	0.502	2	298	0	Straight	1
**2**			4	0.485	3.998	315	2	Straight	1
**3**			6	0.484	5.994	316	6	Straight	1
**4**			8	0.482	7.992	318	8	Straight	1
**5**			10	0.488	9.996	312	4	Straight	1
**6**			20	0.494	19.994	306	6	Straight (Slight Deflection)	0
**7**			40	0.469	39.896	331	104	Deflected	−1
